# Atraumatic Restorative Treatment and Interim Therapeutic Restoration: A Review of the Literature

**DOI:** 10.3390/dj7010028

**Published:** 2019-03-07

**Authors:** Afnan M. Saber, Azza A. El-Housseiny, Najlaa M. Alamoudi

**Affiliations:** 1 Pediatric Dentistry Department, Faculty of Dentistry, King Abdulaziz University, 21589 Jeddah, Saudi Arabia; afnan.saber@gmail.com (A.M.S.); aalhosseiny@kau.edu.sa (A.A.E.-H.); 2 Pediatric Dentistry Department, Faculty of Dentistry, Alexandria University, 21526 Alexandria, Egypt

**Keywords:** atraumatic restorative treatment, interim therapeutic restoration, glass ionomer cement, high-viscosity glass ionomer

## Abstract

This review discusses the techniques and uses of atraumatic restorative treatment (ART) and interim therapeutic restoration (ITR) and states the differences between these two approaches. ART and ITR are similar approaches and are performed using the same material, but they differ in the purpose of their use. ART is used in cases when there are obstacles to reaching dental care units and has been proven to have high success rates in primary and permanent dentitions. ITR is used as a temporary restoration that will be replaced with a more definitive one. ITR is used in cases when the ideal dental treatment cannot be performed. Conventional glass polyalkenoate (ionomer) restorative cement (GIC) is the material of choice that has been used for ART and ITR. This is because of its fluoride release properties, including its ability to bond to enamel and dentine, its pulpal biocompatibility, and its ease of manipulation. High-viscosity glass ionomer performed better than low and medium-viscosity glass ionomer in ART. Combining GIC with conditioner, as well as the use of a chemo-mechanical approach, improved the success rate of ART. Both ATR and ITR are acceptable strategies, with success rates comparable to the traditional treatment methods.

## 1. Introduction

Atraumatic restorative treatment (ART) and interim therapeutic restoration (ITR) have had increased interest in the past few years. ART and ITR are similar approaches and are performed using the same material, but they differ in the purpose of their use [1]. ART is used in cases when there are obstacles to reaching dental care units [1,2] and has been proven to have high success rates in primary dentition [3]. ITR, on the other hand, is used as a temporary restoration that will be replaced with a more definitive one [4].

ART was developed as a treatment approach in developing countries, where resources might not be available for a more definitive treatment [5]. Following this, developed countries began to use the same approach in cases of severe early childhood caries, in order to control the progression of caries through the fluoride-releasing property of the glass ionomers. It was then called ITR [5]. In addition, children that are anxious and difficult to handle in the dental office are suitable candidates for ITR, which has been proven to produce satisfactory results [6].

Conventional glass polyalkenoate (ionomer) restorative cement (GIC) is the material of choice that has been used for ART and ITR. This is because of its fluoride-releasing properties, including its ability to bond to enamel and dentine, its pulpal biocompatibility, and its ease of manipulation [7,8,9,10,11,12,13,14,15]. Moreover, resin-modified glass ionomer cement (RMGIC) performed better than conventional glass ionomer in ART and ITR because of its increased wear resistance [16]. 

Much research has been carried out regarding ART [17]. The indications, technique, materials, and success of ART have been thoroughly examined in various studies [17]. The literature, however, is lacking in research addressing the same aspects but related to ITR. Moreover, not enough articles indicated the differences between the two approaches and why they are named differently. 

The purpose of this review is to discuss the techniques and uses of atraumatic restorative treatment (ART) and interim therapeutic restoration (ITR) and to report the differences between the two approaches.

## 2. Atraumatic Restorative Treatment

### 2.1. Indications for Use

ART is used in cases when routine dental treatment cannot be performed because of a lack of facilities or accessibility to a dental clinic [17]. In addition, ART can be used in schools as a community measure to control caries in a large number of children [18]. ART can be used in both primary and permanent teeth [1].

### 2.2. Definition and Materials

ART is a treatment strategy that requires trained personnel and suitable materials for its success [19,20]. ART is best performed using glass ionomer cement (GIC). GIC (such as Fuji IX, GC Int.) is a glass polyalkenoate cement that consists of calcium or strontium alumino-fluoro-silicate glass powder and water-soluble polymer [21,22]. Several factors led to the selection of GIC as a suitable material for ART. These factors included its fluoride-releasing properties, its ability to bond to enamel and dentine, its pulpal biocompatibility, and its ease of manipulation [7,8,9,10,11].

The fluoride-release from GIC seems to be advantageous for ART. Fluoride that is released from GIC makes the tooth structures (enamel and dentine) more resistant to acidic invasion by bacteria [7,8,23,24,25,26,27,28,29]. Fluoride can be released from glass ionomers for up to five years [24,27]. In addition, GIC acts as a reservoir for fluoride, as it takes up fluoride ions from topical fluoride [28,29]. This property of GIC means that the teeth treated with ART remain less susceptible to caries for long periods [8,21,25,30].

A glass ionomer that is specifically designed for ART is available, which is termed a high-viscosity glass ionomer (such as Ketac Molar Easymix^®^, 3M ESPE, Seefeld, Germany) [18,31,32]. It possesses a high powder-to-liquid ratio, with improved mechanical properties, including wear resistance, compressive strength, and marginal adaptability [18,31]. A high-viscosity glass ionomer is the recommended type of glass ionomer for ART use [33,34]. A high-viscosity glass ionomer is more durable than a low or medium-viscosity glass ionomer [34]. Furthermore, a study performed in 2006 suggested that medium-viscosity glass ionomers should not be used in ART [33]. Different types of GIC are shown in Table 1.

Additionally, silver diamine fluoride (SDF) is a valuable material that can be used in ART [42]. The indications for SDF use include children that are unable to access a dental clinic [42]. SDF provides a minimally invasive approach comprising of the application of the material on the carious dentin without removing the tooth structure [42]. The adverse effects of SDF include discoloration resulting from the silver [42]. The benefits, however, outweigh the risks, thus it is recommended to use SDF in ART [42]. 

Moreover, the hand instruments used in ART have been manipulated to be very sharp, as to make the process of ART faster and to produce some form of cavity preparation [18,31].

### 2.3. Technique of Use

ART is performed by using a sharp hand instrument to remove the carious tooth structure, followed by restoration with GIC or RMGIC [16,43]. The instrument used in ART is a sharp spoon excavator with a diameter of 1 or 1.5 mm to remove the soft caries [44]. In addition, a dental hatchet can be used to widen the cavity [44]. The unaffected fissures should also be sealed by GIC, as a measure of prevention [43]. The ART procedure does not require the use of local anesthetic, as it does not produce pain and it is well accepted by children [18,45].

### 2.4. Success of ART

In 2001, a study was conducted in China regarding the success rate of ART performed on the primary teeth in various cavity designs [46]. They found that, in 30 months of follow-up, the success rates were high for Class I and Class V restorations (79% and 70%, respectively) [46]. For Class II restorations, the success rate was found to be moderate (51%) [46]. However, the success rates for both Class III and Class IV were found to be low [46].

Another study was performed in 2003 that showed that, in 24 months of follow-up, the success rate of ART performed in Class I cavities was high (89.6%) [3]. They also concluded that there was no significant difference in the success rate between ART and amalgam performed in Class I cavities [3].

A meta-analysis was conducted in 2006 which addressed the success rates of ART in primary and permanent dentitions [33]. It concluded that, in 12 months of follow-up, the success rates of ART, made in the surfaces of single teeth and performed using high-viscosity glass ionomer, were 95% and 97% for primary and permanent teeth, respectively [33]. 

Several studies have addressed the success of ART performed in proximal cavities. They all showed that the durability of the GIC placed on the proximal surfaces of teeth treated with ART was significantly lower than the durability of the GIC placed on the occlusal surfaces [47,48,49]. The most common causes of failure were cervical marginal gaps [47], total or partial loss of the restorations [48], and gross marginal defects [48].

Recently, a study was conducted in India addressing the success rate of ATR applied to one or two surfaces [50]. They found that the success rate of ART was comparable to that of composite resins in 12 months of follow-up (89.7% for one surface and 88% for two surface restorations) [50].

Regarding the method of the application of GIC in ART, a study was carried out in Brazil in 2016 which addressed the success rate of ART performed using a bilayer method [51]. It concluded that the bilayer technique of ART increased the survival rate of proximal restorations in primary molars [51].

### 2.5. Combinations

#### 2.5.1. Use with Conditioner

The properties of GIC in ART have been influenced by the use of certain materials. For example, GIC was proven to produce better results when used in conjunction with a dentin conditioner (Cavity conditioner; GC) [44,52]. The conditioner is composed of 20% polyacrylic acid and 3% aluminum chloride hexahydrate [53]. It aids in cleaning the bonding surface of the tooth prior to the use of GIC, by removing the smear layer and debris [53]. Additionally, it has the advantage of sealing the dentinal tubules to eliminate sensitivity [53].

#### 2.5.2. Chemo-Mechanical Approach

The chemo-mechanical method of the removal of caries is a desired method that can be used in ART. The chemo-mechanical approach comprises the use of a chemical material that softens the carious tooth structure, followed by the mechanical removal of caries [54]. This method has the advantages of reducing pain, heat, vibration, and pressure during the treatment, making it accepted by children [55,56].

Several studies have been carried out regarding the chemical material that can be used in the chemo-mechanical removal of caries [57,58,59,60,61,62]. Papacarie and Carisolv are considered the most commonly used materials [57,58,59,60,61,62]. Papacarie is a gel consisting of papain and chloramine [63,64,65]. Papain is an endo-protein that offers bacteriostatic, bactericidal, and anti-inflammatory properties [65]. Chloramine, on the other hand, is formed through the chemical reaction of chlorine and nitrogen, which is derived from ammonia [65]. Chloramine provides bactericidal and disinfectant properties [65]. The additional components of Papacarie include salts, water, toluidine blue, and thickeners [65]. Carisolv is a gel composed of 0.5% sodium hypochlorite and three amino acids, including lysine, leucine, and glutamic acid [66].

Papacarie and Carisolv are used to dissolve carious tooth structures by breaking the collagen fibrils infected by caries, with the ability to preserve sound tissues [56,65]. Therefore, they will facilitate the removal of carious tissues by hand instruments [67]. In ART, Papacarie and Carisolv can be used in conjunction with manual tools, such as hand instruments, for the removal of dental caries [56,63,64]. Since they have the advantage of reducing pain during the treatment, they make ART more acceptable for children [56,63,64,68].

### 2.6. Cost

The ART approach enables the treatment of many children in a community-based setting. This approach is considered economic, as it is performed using simple equipment [69]. However, GIC with lower costs has reduced quality and should not be considered for ART [70,71].

### 2.7. ART in Disabled Patients

Children with disabilities have several barriers to dental treatment [72]. They might have difficulties in coping with the traditional treatment methods performed in the dental chair [72]. In addition, they require proper management by specialized dentists to acquire effective treatment results [72].

A recent study was carried out regarding the effectiveness of ART as an alternative treatment for disabled children [73]. They performed ART with a high-viscosity glass ionomer for 66 children with 16 different types of disabilities [73]. The number of ART procedures performed was 182 [73]. They found that ART is an effective treatment strategy for disabled children [73].

## 3. Interim Therapeutic Restorations

### 3.1. Indications for Use

ITR is indicated in cases where there are obstacles to the performance of an ideal dental treatment [74]. Either this difficulty is related to the cavity preparation or restoration, or to the oral condition of the patient (e.g., early childhood caries) [75]. In both cases, ITR provides a suitable alternative [74].

The indications for the use of ITR include young patients, uncooperative patients, patients with special needs, and cases when traditional dental treatment cannot be performed and needs to be postponed [76,77]. In addition, ITR can be used in step-wise excavation, in partially erupted molars that are difficult to isolate, or in patients with severe caries prior to general anesthesia [78,79]. ITR can be used in both primary and permanent teeth [1].

### 3.2. Definition and Materials

The term ITR was implemented to describe the provisional nature of ITR [80]. ITR is comprised of the same technique as ART and is performed with the same material (GIC) [1]. The use of GIC in ITR has the advantage of reducing the amount of cariogenic bacteria (e.g., Mutans Streptococci, lactobacilli) and helping to neutralize the oral flora [81,82,83]. Teeth treated with ITR should be managed with more definitive restorations within six months of the placement to avoid an elevation in the amount of oral microbes to pre-treatment levels [82].

### 3.3. Technique of Use

The procedure of ITR requires the removal of carious tooth structures using hand or rotary instruments, followed by restoration with GIC or RMGIC [4,84].

### 3.4. Success of ITR

The increased success of ITR was detected in one surface or two small surface restorations [48,85]. The most common cause of the failure of ITR is inadequate cavity preparation, with a subsequent lack of sufficient bulk and compromised retention [48,86].

Generally, ITR should be replaced with a more definitive restoration within six months of the placement to ensure maximum benefit to the patient and to reduce the risk of failure, as the levels of oral cariogenic bacteria might return to pre-treatment levels after six months of treatment [82].

### 3.5. ITR for Managing Early Childhood Caries (ECC)

In 2016, a study was carried out comparing the success of ITR with conventional drilling treatment methods in the treatment of early childhood caries (ECC) [75]. It was found that the number of teeth which were considered to have failed was significantly higher in the ITR group than in the conventional method group [75]. The conclusion, however, stated that ITR is a suitable treatment approach for the management of ECC, as it controls the progression of caries and allows more children to be treated [75]. 

### 3.6. ITR versus General Anesthesia

In a recent study, ITR was compared with general anesthesia for managing young uncooperative patients [6]. The study results illustrated that ITR has higher risks of treatment failures than general anesthesia [6]. However, it is still an acceptable method and can be beneficial when used in certain cases in the dental clinic [6].

## 4. ART versus ITR

Despite the similarities between ART and ITR, there are certain aspects that make the two approaches different. In most of the cases that ART has been used, there was no plan to replace it with a more definitive treatment. This is because the indications for its use stated that it was used when there were obstacles to reaching dental care units. Therefore, there were also most probably obstacles to replacing it with a more definitive treatment [17]. ART is performed in areas lacking facilities and is often mistakenly interpreted as a permanent restoration [4].

On the other hand, ITR was developed as a temporary approach that would be replaced with a more definitive restoration, and hence it is named ITR. ITR should be replaced with a more definitive restoration within six months of the placement to ensure its maximum benefit is attained and to reduce the risk of failure [82].

## 5. Conclusions

Based on the available literature, we conclude that ATR and ITR are suitable treatment approaches for the management of dental caries in several conditions in both primary and permanent teeth. ART and ITR are similar approaches and are performed using the same material (GIC). 

ATR is used in cases where there are obstacles to reaching the dental care units. ITR, on the other hand, is used for treating patients in dental clinics in order to control the progression of caries or to manage certain health characteristics of the patient. 

A high-viscosity glass ionomer performed better than low and medium-viscosity glass ionomers in ART. Combining GIC with conditioner, as well as the use of the chemo-mechanical approach, improved the success rate of ART.

Both ATR and ITR are acceptable strategies, with success rates comparable to the traditional treatment methods. Finally, the literature is deficient in studies on ITR, therefore, more studies should be carried out regarding this treatment approach.

## Figures and Tables

**Table 1:** The different types of glass ionomer cement (GIC) used for atraumatic restorative treatment (ART).

Material	Manufacturer	Viscosity	Duration
ChemFil	Dentsply	Low [35]	24 months [36]
ChemFlex	Dentsply	Low	12 months [37]
Fuji IX	GC Int.	High [35]	6–12 months [22,38,39]
Fuji Plus	GC Int.	Low	6 months [38]
Ketac-Molar	ESPE GmbH	High [35]	12–30 months [40,41]

## Abbreviations

ART	atraumatic restorative treatment
ITR	interim therapeutic restoration
GIC	glass ionomer cement
ECC	early childhood caries
